# Transarterial embolization with bleomycin–lipiodol for symptomatic multifocal hepatic hemangiomas: Concurrent nontarget lesion devascularization via permitted reflux

**DOI:** 10.1016/j.radcr.2026.07.085

**Published:** 2026-08-22

**Authors:** Tsz King Donald Chow, Joel Jihwan Hwang, Ali Malik

**Affiliations:** a Department of Interventional Radiology, Saint Louis University School of Medicine, St. Louis, MO 63104, USA; b Department of Radiology, Saint Louis University School of Medicine, St. Louis, MO 63110, USA

**Keywords:** Hepatic hemangioma, Transarterial chemoembolization, Bleomycin-lipiodol, Multifocal liver hemangioma, Symptomatic hemangioma, TACE

## Abstract

Hepatic hemangiomas are the most common benign liver tumors and are typically managed conservatively. Larger or symptomatic lesions may require intervention. Transarterial chemoembolization with bleomycin-lipiodol is a minimally invasive alternative to surgery, offering symptom relief and a favorable safety profile. The therapeutic reach of bleomycin-based transarterial chemoembolization in multifocal hepatic hemangioma—including its effects on nontarget lesions—remains under investigation. A 41-year-old female presented with 2 months of progressive right upper quadrant pain refractory to analgesics. Computed tomography demonstrated 2 right hepatic hemangiomas. The patient underwent transarterial chemoembolization with bleomycin–lipiodol targeting the dominant subdiaphragmatic lesion. During super-selective embolization of the dominant lesion, a limited degree of reflux into an adjacent arterial branch supplying the inferior lesion was permitted, resulting in angiographic devascularization of both lesions. After treatment, the patient experienced complete symptomatic resolution at 7 months. Serial computed tomography demonstrated sustained treatment response, with estimated lesion volume reductions of approximately 72% (dominant) and 58% (inferior) using the ellipsoid approximation. This case supports bleomycin–lipiodol transarterial chemoembolization for symptomatic hepatic hemangiomas and contributes to growing evidence that bleomycin-based transarterial chemoembolization may be associated with size reduction of nontargeted lesions. However, the mechanism underlying this finding cannot be established from a single case, and the relative contributions of permitted reflux, shared arterial supply, and the systemic effects of bleomycin-based TACE to the observed size reduction of the nontarget lesion remain undetermined. Transarterial chemoembolization represents a compelling option for symptomatic hepatic hemangiomas, particularly where surgical complications are high.

## Introduction

Hepatic hemangiomas are the most common benign liver tumors, with a prevalence of up to 8% in the general population [1]. Although the vast majority of hepatic hemangiomas are asymptomatic and managed conservatively, larger lesions (≥5 cm) may produce symptoms such as right upper quadrant pain, abdominal fullness, and early satiety secondary to mass effect [[2], [3], [4], [5]]. Beyond symptomatic burden, spontaneous rupture is an extremely rare but life-threatening complication, particularly for larger hepatic hemangiomas. The overall incidence of rupture is approximately 0.47% across all hepatic hemangiomas and 3.2% among giant hemangiomas. Additional risk factors include exophytic location and pregnancy [3]. Multiphasic contrast-enhanced computed tomography (CT) is integral to diagnosis, with hemangiomas typically demonstrating peripheral nodular enhancement on arterial phase imaging followed by complete centripetal fill-in on delayed phases [2,6]. However, diagnostic confidence may be reduced in patients with chronic hepatitis B, cirrhosis, or known malignancies [7]. Beyond diagnosis, imaging also provides useful information regarding the lesion and hepatic arterial anatomy, which aids in surgical and interventional planning.

Surgical resection has historically been the definitive therapy for large and symptomatic hemangiomas, although there are associated procedural complications such as bleeding, especially for larger lesions [8]. Transarterial chemoembolization (TACE) has emerged as a minimally invasive alternative that selectively targets and occludes the arterial supply of hemangiomas via various embolization agents [[9], [10], [11]]. More specifically, TACE with bleomycin-lipiodol works by combining a sclerosing agent with a viscous carrier that enhances the accumulation of drugs in the desired hepatic region. Bleomycin induces endothelial injury within the cavernous vascular spaces, while lipiodol facilitates prolonged contact with the lesion [[12], [13], [14]]. Previous systematic review and meta-analysis have shown that lipiodol-based TACE procedures were generally safe and associated with effective symptomatic relief and hemangioma size reduction [11,15]. Despite increasing use of TACE, most reported cases describe treatment of single lesions. The progression and outcome of multifocal hemangiomas treated with embolization, particularly in the setting of shared arterial supply, remain incompletely studied.

We present a case of symptomatic multifocal hepatic hemangiomas treated with a single session of bleomycin–lipiodol TACE directed toward the dominant lesion. During super-selective embolization of the dominant hemangioma, intentional reflux was permitted into adjacent arterial branches supplying a second hemangioma, resulting in angiographic devascularization of both hemangiomas. Both hemangiomas subsequently demonstrated interval size reduction on follow-up imaging, and the patient experienced symptomatic improvement. This case highlights a unique procedural observation and raises questions regarding the relative contributions of direct embolization, shared vascular anatomy, and potential systemic bleomycin exposure to the observed regression of both hemangiomas.

## Case presentation

A 41-year-old female with a history of known hepatic hemangiomas and hypertension presented with 2 months of progressive right upper quadrant pain. She also complained of a constant pressure-like sensation radiating to the back, which worsened at night and was disruptive to sleep. Associated symptoms included decreased appetite and nausea. The pain was unresponsive to tramadol. Physical examination revealed tenderness to palpation in the right upper quadrant. Laboratory evaluation, including complete blood count, comprehensive metabolic panel, and hepatic function panel, was within normal limits.

**Fig. 1:**
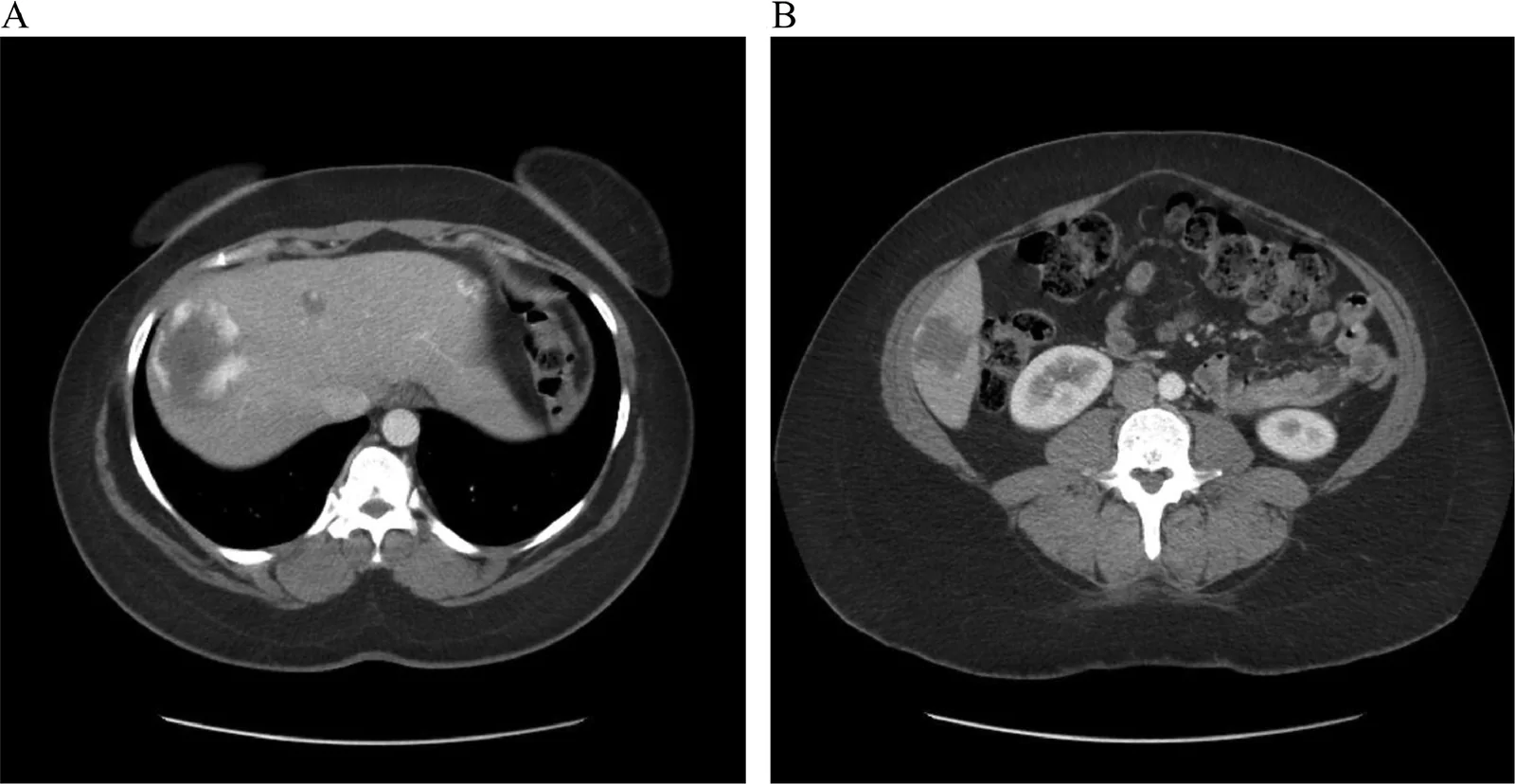
Axial CT findings of multifocal hepatic hemangiomas. (A) Contrast-enhanced CT demonstrates a subdiaphragmatic hypodense lesion with peripheral contrast enhancement in segment 7/8 measuring 5.1 × 5.9 cm on the axial view (craniocaudal diameter 6.4 cm, measured on the sagittal view; not shown). (B) Contrast-enhanced CT demonstrates an inferior hypodense lesion with peripheral contrast enhancement in segment 6 measuring 3.0 × 3.5 cm on the axial view (craniocaudal diameter 3.4 cm, measured on the sagittal view; not shown).

Contrast-enhanced CT of the abdomen and pelvis demonstrated multiple hypodense hepatic lesions with characteristic peripheral contrast enhancement, consistent with multifocal hepatic hemangiomas (Fig. 1A and B). No intrahepatic biliary dilatation or other acute intra-abdominal pathology was identified. Given the multifocal disease nature, refractory symptoms despite pharmacologic therapy, and the patient’s preference against surgery, a decision was made to proceed with TACE. The initial treatment plan was to target the larger lesion in hepatic segment 7/8, given its capsular involvement and likely contribution to the patient’s pain via mass effect.

## Procedure

**Fig. 2:**
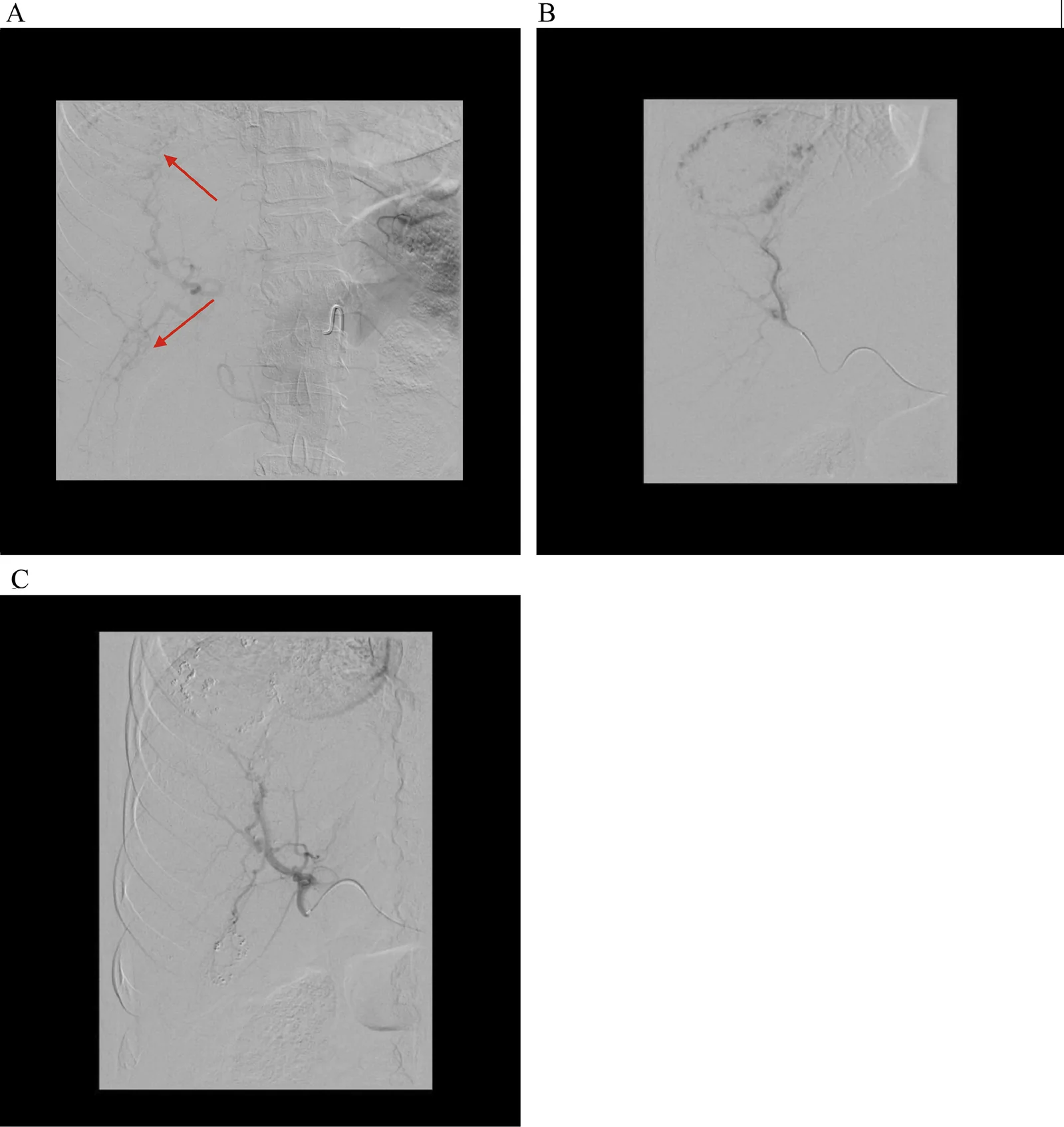
Angiogram of hepatic hemangiomas before and after embolization. (A) Angiogram after catheterization of the celiac artery, demonstrating characteristic pooling of contrast and peripheral staining of the subdiaphragmatic hemangioma and inferior hemangioma (indicated by the 2 red arrows). (B) Angiogram after selective catheterization of the superior branch of the right hepatic artery, demonstrating characteristic pooling of contrast in the superior hemangioma. (C) Postembolization angiogram shows satisfactory stasis and devascularization of both hemangiomas.

Right common femoral artery access was obtained under ultrasound guidance, and a 5 French vascular sheath was placed. A 5 French SOS catheter was used to catheterize the celiac artery. Angiography demonstrated normal hepatic arterial anatomy, including a cranially oriented branch of the right hepatic artery extending towards the hepatic dome, near the region of the patient’s subdiaphragmatic hemangioma. Angiography also demonstrated 2 hepatic hemangiomas with contrast pooling, consistent with previous CT findings (Fig. 2A).

A 2.4 French Progreat microcatheter was utilized to gain access into the right hepatic artery and subsequently into the superior branch supplying the subdiaphragmatic hemangioma, which had been designated as the primary target lesion based on preprocedure planning. Selective angiography demonstrated contrast pooling and the peripheral staining of the subdiaphragmatic hemangioma (Fig. 2B).

**Fig. 3:**
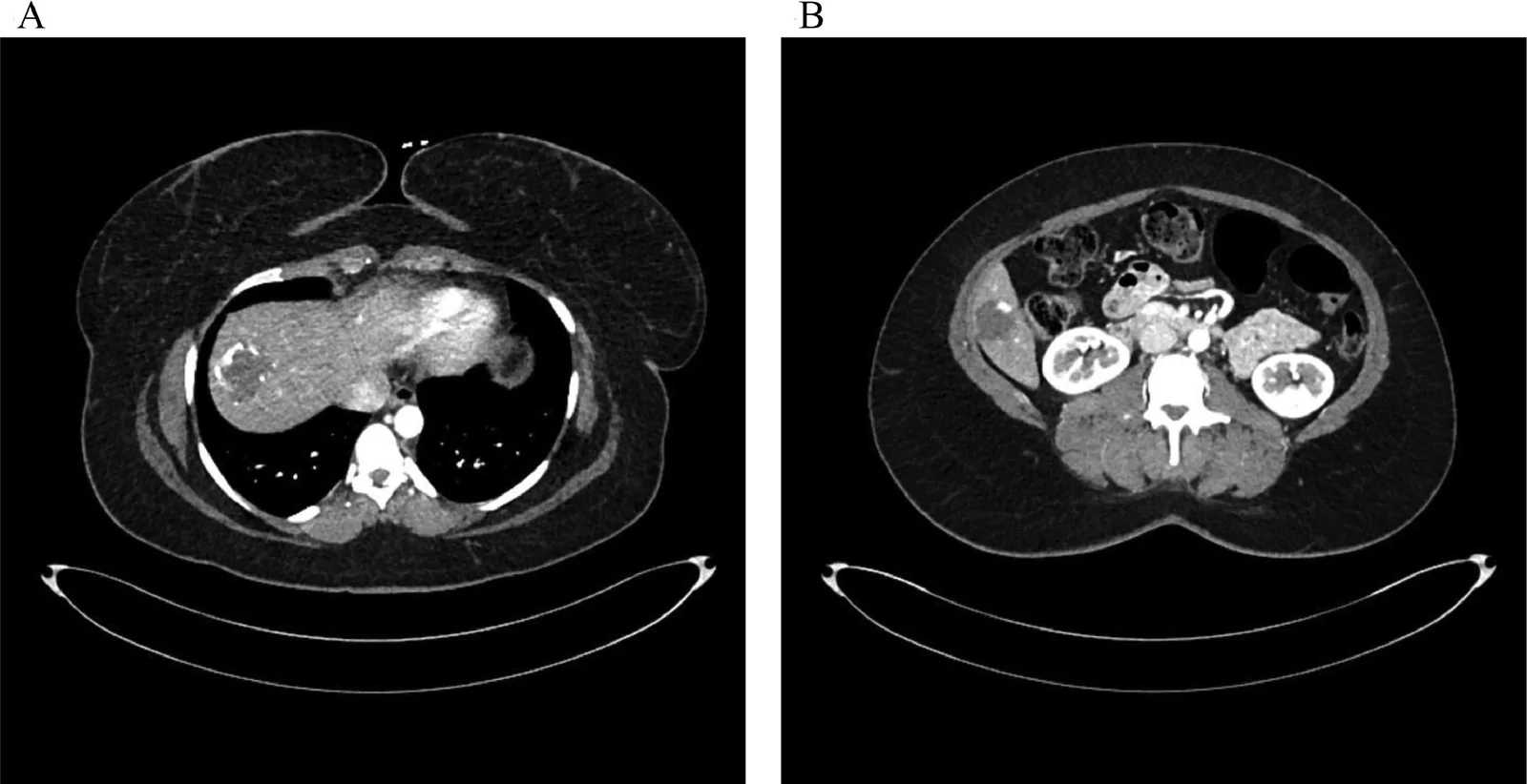
Axial CT findings of multifocal hepatic hemangiomas after a single session of embolization. (A) CT abdomen multi-phase W contrast demonstrates a 3.9 cm × 3.6 cm lesion on the axial view (craniocaudal diameter 3.8 cm, measured on sagittal view; not shown) in segment 7/8 with nodular, discontinuous peripheral enhancement, likely representing a hemangioma status post treatment changes. Pretreatment lesion measured 5.1 × 5.9 cm × 6.4 cm. (B) CT abdomen multi-phase W contrast demonstrates a 2.3 cm × 2.4 cm lesion on the axial view (craniocaudal diameter 2.7 cm, measured on sagittal view; not shown) in segment 6 with nodular, discontinuous peripheral enhancement, likely representing a hemangioma status post treatment changes. Pretreatment lesion measured 3.0 × 3.5 cm × 3.4 cm.

Embolization was performed using a bleomycin–lipiodol mixture (approximately 11 mL of mixture containing 30 units of bleomycin). Toward the end of super-selective embolization of the segment 7/8 lesion, limited reflux of embolic material into the adjacent right hepatic arterial branches supplying the segment 6 hemangioma was intentionally permitted while carefully monitoring for nontarget embolization outside the intended hepatic arterial distribution. Angiography after embolization demonstrated satisfactory stasis with devascularization of both segment 7/8 and 6 lesions (Fig. 2C). Because both lesions already demonstrated satisfactory angiographic devascularization, an additional super-selective embolization of the segment 6 lesion was not performed, thereby avoiding unnecessary catheter manipulation and the potential risk of nontarget reflux. Hemostasis of the access site was achieved using a Mynx closure device. Outpatient follow-up imaging was planned to assess for interval hemangioma regression and clinical response (Table 1 and Fig. 3A).

**Table 1:** Clinical and radiographic follow-up after a single session of embolization.

Timepoint	Key findings
December 2025 (3 mo postprocedure)	Near-complete resolution of daytime pain; nocturnal pain reduced to 3/10 intermittently. CT: Segment 7/8 decreased to 3.9 × 3.6 cm × 3.8 cm; Segment 6 decreased to 2.3 × 2.4 cm × 2.7 cm, corresponding to estimated volumetric reductions of approximately 72% and 58%, respectively, using the ellipsoid approximation (V = π/6 × L × W × H). Post-treatment changes confirmed in both lesions (Fig. 3A and B).
April 2026 (7 mo postprocedure)	Fully asymptomatic—pressure sensation and pain completely resolved. CT: Segment 7/8 hemangioma continued to demonstrate marked size reduction compared with baseline. Stable postembolization changes in both lesions.

## Discussion

This case demonstrates both clinical and radiographic response following a single session of bleomycin–lipiodol TACE for a patient with symptomatic multifocal hepatic hemangiomas. Consistent with previous studies demonstrating high rates of symptom improvement following lipiodol-based TACE, our patient achieved complete resolution of symptoms by 7 months [15]. A recent meta-analysis of 13 TACE studies involving 1586 patients with hepatic hemangioma also reported a clinical success rate of 99.9%, defined as symptom relief without additional intervention [16]. Moreover, there were no postprocedural complications observed in our patient, consistent with the favorable safety profile reported in large meta-analyses [11]. The most common minor complications following TACE for hepatic hemangioma are postembolization syndrome and transient liver enzyme elevation, occurring in approximately 36% and 37% of lipiodol-based procedures, respectively [15]. Postembolization syndrome, characterized by abdominal pain, fever, nausea, and vomiting, is typically self-limited. While rare, complications such as liver abscess, ischemic cholecystitis from nontarget cystic artery embolization, and hemoglobin drop requiring packed red blood cell transfusion have also been reported across studies [15]. Regarding bleomycin related complications, although bleomycin carries a well-known risk of pulmonary toxicity, particularly at cumulative systemic doses exceeding 300 units, the doses used in hepatic hemangioma embolization are substantially lower [13,17]. To date, no cases of bleomycin-induced pulmonary toxicity have been reported across the hepatic hemangioma TACE literature [11,13,18]. However, systemic absorption of bleomycin may occur following bleomycin-based TACE for hepatic hemangiomas, and pulmonary outcomes have not been a primary focus in most studies [19].

Hepatic hemangiomas derive their blood supply predominantly from the hepatic arterial system, enabling selective embolization while preserving surrounding parenchyma [20]. In this patient, super-selective catheterization of the superior branch of the right hepatic artery enabled targeted embolization of the dominant hemangioma. Toward the end of embolization, limited reflux into adjacent arterial branches supplying the segment 6 hemangioma was intentionally permitted, resulting in angiographic devascularization of both hemangiomas. Importantly, the reflux was recognized during the procedure and deliberately permitted rather than occurring inadvertently. This procedural observation highlights the importance of careful angiographic evaluation and vascular mapping when treating multifocal hepatic hemangiomas, especially when adjacent lesions share arterial territories that may influence embolic distribution during TACE. Existing studies on hepatic hemangioma have focused on identifying and mapping the primary arterial supply to individual lesions, with limited studies directly evaluating surrounding vascular connections between separate lesions [21,22].

The CT findings at 3 months postprocedural follow-up were notable for a substantial reduction in hemangioma size. Using the ellipsoid approximation (V = π/6 × L × W × H), the estimated lesion volumes decreased by approximately 72% (segment 7/8, from ∼100.8 to ∼27.9 cm³) and 58% (segment 6, from ∼18.7 to ∼7.8 cm³). The 2 in-plane (width and length) diameters were measured on the axial view, and the craniocaudal diameter was measured on the sagittal view. These findings are consistent with previous reported outcomes following bleomycin–lipiodol TACE for hepatic hemangiomas. A large single-center retrospective study of 121 patients demonstrated a mean maximum hemangioma diameter decrease from 122 mm to 73 mm after treatment [18]. Similarly, another retrospective study of 31 patients reported a clinical success rate of 80.6%, defined as a volume reduction of 50% or more on follow-up imaging [22]. Furthermore, 1 systematic review and meta-analysis comparing lipiodol-based and nonlipiodol-based TACE techniques found significantly greater hemangioma size reduction with lipiodol-based approaches [15].

An important point of discussion is the degree of size reduction observed in the nontargeted hemangioma for our patient. The approximately 58% reduction in estimated volume is difficult to attribute to any single mechanism. Because the amount of embolic material reaching the second lesion could not be quantified, it is not possible to determine the relative contributions of direct embolization through intentional reflux, shared arterial supply, or other mechanisms. One additional explanation that has been proposed in the literature is that bleomycin-based TACE may exert therapeutic effects beyond the directly embolized arterial territory. In the study by Kutlu et al., 24 patients with giant hepatic hemangiomas underwent selective bleomycin–ethiodized oil TACE. The authors reported a significant size reduction of untreated nontarget hemangiomas, with 53% of nontarget lesions showing ≥50% volume reduction at follow-up. More importantly, the authors proposed 2 speculative mechanisms for the observed nontarget hemangioma regression. The first is diffusion of bleomycin through the systemic circulation, exposing untreated lesions to therapeutic concentrations. The second is a locoregional effect triggering immune-mediated responses or angiogenic factor modulation at adjacent sites. However, both mechanisms remain unproven, and further pharmacokinetic studies are needed to confirm these hypotheses [19]. The present case contributes additional observational evidence that bleomycin–lipiodol TACE may have a broader therapeutic reach beyond the directly embolized lesion in multifocal hepatic hemangioma disease, necessitating further investigation into this matter for treatment optimization.

Although follow-up in this case is limited to 7 months, long-term outcomes of lipiodol-based TACE have generally been favorable. One study demonstrated sustained lesion reduction in patients followed for at least 36 months, while another reported greater than 50% size reduction in the majority of patients with follow-up extending to 60 months [18,21]. Moreover, successful long-term results beyond 5 years following superselective TACE in symptomatic hemangiomas have been described [23,24].

This case also highlights the role of TACE as a minimally invasive alternative to surgical management in the setting of symptomatic hepatic hemangioma. Current guidelines consider TACE as an alternative to surgery for complicated hemangiomas [7]. However, the high clinical success rates and low complication rates are increasingly making TACE a more favorable and viable treatment option, especially in cases of giant and multifocal hemangiomas where surgical complications are high [7,11]. It is important to note that no randomized controlled trials exist to definitively compare the 2 approaches; thus, early referral for Interventional Radiology evaluation may therefore be appropriate in patients who fail conservative management or are poor surgical candidates [7].

## Limitations

This case has several important limitations. First, as a single case report, generalizability is limited, and causal relationships cannot be established. Second, although both lesions demonstrated angiographic devascularization during the procedure and shrinkage on follow-up CT, the relative contributions of intentional embolic reflux, shared arterial anatomy, and potential systemic bleomycin exposure cannot be determined. Furthermore, the amount of embolic material delivered to each lesion cannot be precisely quantified. Third, lesion volumes were estimated using the ellipsoid approximation derived from maximal orthogonal measurements rather than dedicated volumetric analysis software, which may overestimate or underestimate changes in lesion volume following treatment. Fourth, symptom improvement in this case was assessed using patient-reported outcomes rather than a validated or standardized scoring instrument. As a result, the degree of clinical improvement could not be objectively quantified. Finally, follow-up was limited to 7 months, precluding assessment of the durability of treatment response and potential for hemangioma recurrence. Long-term follow-up is necessary to confirm sustained response and evaluate for recurrence.

## Conclusion

This case demonstrates that bleomycin–lipiodol TACE can achieve clinical and radiographic response in symptomatic multifocal hepatic hemangiomas. In the present case, the concurrent size reduction of both the targeted and nontargeted hemangiomas, following embolization of the intended target lesion and permitted reflux into the nontargeted hemangioma, raises important questions regarding the relative contributions of intentional embolic reflux, shared arterial supply, and potential regional or systemic effects of bleomycin-based TACE. Although this pattern of lesion response is consistent with emerging reports of nontarget hemangioma regression after bleomycin-based TACE, it should be interpreted as hypothesis-generating rather than as evidence favoring any single therapeutic mechanism. Given its favorable safety profile and high clinical success rates, TACE represents a minimally invasive option for symptomatic hepatic hemangiomas.

## Patient consent

Written informed consent for publication of this case report and accompanying images was obtained from the patient.
